# Overexpression of SlGATA17 Promotes Drought Tolerance in Transgenic Tomato Plants by Enhancing Activation of the Phenylpropanoid Biosynthetic Pathway

**DOI:** 10.3389/fpls.2021.634888

**Published:** 2021-03-16

**Authors:** Tingting Zhao, Tairu Wu, Tong Pei, Ziyu Wang, Huanhuan Yang, Jingbin Jiang, He Zhang, Xiuling Chen, Jingfu Li, Xiangyang Xu

**Affiliations:** ^1^Laboratory of Genetic Breeding in Tomato, College of Horticulture and Landscape Architecture, Northeast Agricultural University, Harbin, China; ^2^Key Laboratory of Biology and Genetic Improvement of Horticultural Crops (Northeast Region), Ministry of Agriculture and Rural Affairs, Northeast Agricultural University, Harbin, China

**Keywords:** GATA transcription factors, tomato, drought stress, phenylpropanoid biosynthesis, overexpression

## Abstract

GATA transcription factors (TFs) are widely distributed in eukaryotes. Some GATA TFs have been shown to be related to photosynthesis, germination, circadian rhythm, and other functions in plants. Our previous study found that some members of this family have obvious responses when tomato plants are subjected to drought stress, in which the *SlGATA17* gene is significantly upregulated. To further verify the function of this gene under drought stress, we constructed tomato lines with this gene overexpressed. Phenotypic and physiological indicators indicated that the *SlGATA17-*overexpressing plants were more drought tolerant than the wild-type plants. Transcriptomic sequencing results showed that the overexpression of the *SlGATA17* gene improved the activity of the phenylpropanoid biosynthesis pathway. The PAL enzyme activity assay results confirmed that the initial activity of this pathway was enhanced in transgenic plants, especially in the initial response stage, indicating that the *SlGATA17* gene regulates the drought resistance of tomato plants by regulating the activity of the phenylpropanoid biosynthesis pathway.

## Introduction

Tomato (*Solanum lycopersicum* L.) is one of the most widely cultivated and economically important crop plants worldwide. Tomato is also a model system in plant research. Because tomatoes are sensitive to drought stress, they need sufficient water to grow. For example, drought will lead to a decrease in tomato yield, an increase in disease, and a decrease in fruit quality. Therefore, improvement in the drought resistance of tomato varieties is important. Transcription factors (TFs) are proteins that bind to DNA-regulatory sequences to modulate the rate of gene transcription, which plays an important role in plant growth and stress regulation. Studies have shown that multiple family TFs are involved in drought resistance regulation in tomato. A study has shown that JUNGBRUNNEN1, a TF of the NAC family, enhances drought tolerance in tomato (Thirumalaikumar et al., 2018). Zhao et al. found that downregulating the expression of the ZF-HD TF, *SL-ZH13*, decreases the drought tolerance of tomato (Zhao et al., 2019). The heat-shock TF, *HsfA1a*, plays a positive role in the induction of autophagy under drought stress in tomato tolerance to drought stress (Wang et al., 2015).

GATA TFs are major transcriptional regulators that are widely distributed in eukaryotes (Nutan et al., 2020). GATA TFs play important roles in many developmental processes and regulate gene transcription by binding to the (A/T)GATA(A/G) consensus sequence (Reyes et al., 2004). The DNA-binding domain of GATA TFs is constituted by one or two type IV zinc fingers in the form of CX2CX17–20CX2C (C, cysteine; X, any residue) followed by a highly basic amino acid stretch (Lowry and Atchley, 2000). Genes of the GATA family have been identified in several species as follows: 64 GATA genes in soybeans (Zhang et al., 2015); 29 and 28 GATA genes in *Arabidopsis* and rice, respectively (Reyes et al., 2004); 39 putative GATA genes in *Populus* (An et al., 2020); and 30 GATA genes in tomato (Yuan et al., 2018).

The GATA genes have many regulatory functions. In *Arabidopsis*, the GATA zinc finger protein, CONSTANS (Putterill et al., 1995), and its homologous genes in rice (Song et al., 1998) and perennial ryegrass (Martin et al., 2004) are related to photoperiodic control of flowering. Previous observations have shown that GATA motifs are enriched in promoters of genes controlled by circadian rhythms and light-regulated genes (Argüello-Astorga and Herrera-Estrella, 1998). According to Berger et al. (2006), *AreA* from the fungus *Aspergillus nidulans* is a key GATA regulator of nitrogen signaling. Liu et al. (2005) indicated that the GATA zinc finger protein, BME3, is a positive regulator of seed germination in *Arabidopsis* seeds. In addition, one study has demonstrated that the GATA TF, *TaGATA1*, is also associated with biotic stress. It has been shown that overexpression of *TaGATA1* significantly enhances the resistance of wheat to *Rhizoctonia cerealis*, whereas silencing *TaGATA1* suppresses the resistance (Liu et al., 2020). Thus, these findings indicate that GATA genes play an essential role in a wide array of biological processes.

In our previous study, systematic bioinformatics analysis was conducted on 30 members of the GATA family in the tomato genome. By analyzing the stress response patterns of the expression of these genes, we found that some of the genes in this family responded significantly to drought stress, especially the *SlGATA17* gene. The expression of *SlGATA17* was rapidly upregulated in the early stage under drought stress (Yuan et al., 2018). However, no genetic evidence for the roles of GATA TFs in drought responses has been reported in plants. Therefore, a *SlGATA17* tomato transgenic line was constructed in this study to further verify the function of this gene in the regulation of drought resistance in tomato and to elucidate its regulatory pathway. We verified the drought-resistant ability of this gene and found the pathways of differential response regulated by *SlGATA17* transcriptomic sequencing analysis. This work laid the foundation for the study of the precise regulatory mechanism of this gene and provided a reference for the breeding of resistant tomato varieties.

## Materials and Methods

### Plant Materials and Treatment

The “Micro-Tom” tomato cultivar used for the *Agrobacterium tumefaciens*–mediated transformation experiment was obtained from the tomato Research Institute (Northeast Agricultural University, Harbin, China). Tomato plants, including the transgenic tomato lines and wild-type (WT) tomato plants, were grown on soil in a phytotron with 16-h light/8-h dark cycles and 60% relative humidity at 28 and 20°C, respectively. Three WT plants were sprayed with 100 mg/L, 0.1 mmol/L, and 0.4 mmol/L solutions of abscisic acid (ABA), jasmonic acid (JA), and salicylic acid (SA), respectively, and *SlGATA17* expression was then measured at different time points after treatment using quantitative reverse transcriptase–polymerase chain reaction (qRT-PCR). Plants at the 5–8-leaf stage were subjected to drought stress. Plants were removed from the soil, and roots were cleaned. Then, roots were soaked in PEG 6,000 solution (15% PEG solution). Leaves were collected and frozen in liquid nitrogen at different time points (0, 3, and 6 h) after treatment. Three biological replicates were performed for each time point.

### Gene Cloning and Phylogenetic Analysis of the *SlGATA17* Gene Family

Total RNA was extracted from sampled leaves of Micro-Tom seedlings using TRIzol reagent (TRIzol; Invitrogen, Shanghai, China) according to the manufacturer’s instructions. First-strand cDNA was synthesized using a RevertAid First Strand cDNA Synthesis Kit (Thermo Fisher scientific, Waltham, MA, United States) according to the manufacturer’s instructions. The target sequence of the DNA was amplified with specific primers designed by Primer 5.0 software. After amplification, the cDNA fragment was cloned into the digested vector and sequenced by Beijing Genomics Institution (BGI). The protein structure was analyzed using SMART^1^. All GATA gene sequences were downloaded from the NCBI database, and the GATA family phylogenetic relationships of all GATA genes in tomato were analyzed using the NJ method with bootstrapping analysis (1,000 replicates).

### Subcellular Localization of *SlGATA17*

The cDNA of *SlGATA17* (Solyc05g056120.2.1) without the stop codon was amplified using the *SlGATA17*–green fluorescent protein (GFP)-F/*SlGATA17*-GFP-R primer pair (Supplementary Table S1) and cloned into the pCAMBIA2300-GFP vector under the control of the 35S promoter to produce *SlGATA17*-GFP by *Sma*I and *Sal*I restriction digestion. Leaves of *Nicotiana benthamiana* plants were infiltrated with the *Agrobacterium* strain, GV3101, with a recombinant plasmid containing a GFP fusion gene and a control plasmid containing GFP alone. GFP fluorescence expressed in epidermal cells was detected using a confocal microscope (Leica TCS SP8, Germany) at 36 h after infiltration.

### Transcriptional Activation Analysis in Yeast

*SlGATA17* transactivation was investigated using a yeast system. The complete CDS, segments A and B of *SlGATA17* (Solyc05g056120.2.1), were amplified by the *SlGATA17*-F/R, A-*SlGATA17*-F/R, and B-*SlGATA17*-F/R primers (Supplementary Table S1), which were tailed with *Bam*HI and *Eco*RI restriction sites, and ligated into the pGBKT7 vector to generate the *SlGATA17-*pGBKT7 constructs. pGBKT7-53 + pGADT7-T, as a positive control, and pGBKT7-Lam + pGADT7-T, as a negative control, were transformed into the Y2Hgold yeast strain. These transformants were then grown on SD/–Leu/–Trp/–His with X-α-Gal medium at 30°C for 3 days.

### Generation of Transgenic Plants

The full-length cDNA of *SlGATA17* (Solyc05g056120.2.1) was cloned with the *SlGATA17*-F/*SlGATA17*-R primers. The amplified products were digested and cloned into the pCAMBIA2300 plant binary vector to construct an overexpression vector. Transgenic plants were generated by previously published *Agrobacterium*-mediated transformation methods (Van Eck et al., 2006). The transgenic lines were screened through kanamycin selection and validated by a qRT-PCR assay. The *SlGATA17* primers used for qRT-PCR are shown in Supplementary Table S2.

### Physiological Index Measurement

To visualize reactive oxygen species (ROS) accumulation in plants, a nitroblue tetrazolium (NBT) assay was performed according to Rao and Davis (1999), and 3,3′-diaminobenzidine (DAB) staining was performed according to Ramel et al. (2009). We measured the weight of fresh leaves of each sample before drying (W1) and the weight of dried leaves of each sample (W2). The moisture content was calculated using the following formula: moisture content (%) = $\frac{\mathrm{W1}-\mathrm{W2}}{\mathrm{W1}}\times 100$. SOD activity was measured using the SOD test kit (SOD-1-Y, Comin, China) and the POD test kit (POD-1-Y, Comin, China). MDA and Pro contents were measured using the MDA test kit (MDA-1-Y, Comin, China) and Pro test kit (PRO-1-Y, Comin, China), respectively. Three biological replicates were performed.

### RNA Isolation and qRT-PCR Analysis

Total RNA for the qRT-PCR assay was extracted using TRIzol reagent (Life Technologies). First-strand cDNA was synthesized using the M-MLVRTase cDNA synthesis kit (Takara, Dalian, China) according to the manufacturer’s instructions. qRT-PCR was performed using the ChamQ Universal SYBR qPCR Master Mix (Vazyme, Nanjing, China) and the iQ5 system. The 2^–ΔΔCT^ method was used to calculate the relative gene expression (Livak and Schmittgen, 2001). The tomato *EFα1* gene was used for normalization.

### cDNA Library Construction and Sequencing

Samples of WT and both overexpression lines were collected at 0, 3, and 6 h under drought stress and used for RNA-seq analysis. Samples from overexpression lines were mixed for sequencing. The extracted total RNA was sent to BGI (Shenzhen, China) for high-throughput RNA sequencing. First, mRNA with an incorporated polyA tail was enriched with oligo-dT magnetic beads. The obtained mRNA was synthesized into double-stranded cDNA, which was then purified and recovered. The 3′ end of the cDNA was added with a base “A” and adapters. Finally, PCR amplification was performed. Thus, a total of six libraries were constructed, and each library was represented by three biological replications. The libraries were sequenced using paired end Illumina (HiSeq 4,000) sequencing technology.

### RNA Sequencing Data Processing, Differentially Expressed Gene Identification, Gene Ontology Analysis, and Kyoto Encyclopedia of Genes and Genomes Analysis

Clean reads were obtained by removing reads containing adapters, and reads with unknown base content greater than 5% and low-quality reads were removed from raw data. The high-quality reads were aligned to the annotated *S. lycopersicum* reference genome using hierarchical indexing for spliced alignment of transcripts (Kim et al., 2015).

Differential expression analysis to identify the differentially expressed genes (DEGs) was performed using DEseq2 (Li and Dewey, 2011). Genes with an adjusted *P* < 0.05 and an absolute value of log2 ≥ 1 found by DESeq were assigned as differentially expressed. Gene Ontology (GO) enrichment analysis of the DEGs was implemented by the GOseq R package. KOBAS software was used to test the enrichment of DEGs in Kyoto Encyclopedia of Genes and Genomes (KEGG) pathways (Mao et al., 2005). The data generated for this study can be found in the NCBI database, and the GEO accession number is GSE148530.

### Validation of Gene Expression Patterns in the Transcriptome by qRT-PCR Analysis

Fifteen DEGs in the transcriptome were randomly selected, and their expression patterns were verified by qRT-PCR with the method described above. The primers are shown in Supplementary Table S2.

### Measurements of Phenylalanine Ammonia Lyase Activity

Phenylalanine ammonia lyase (PAL) activity was measured using a PAL test kit (PAL-1-Y, Comin, China). PAL catalyzes L-phenylalanine decomposition into *trans*-cinnamic acid and ammonia. *trans*-Cinnamic acid had the maximum absorption value at 290 nm. PAL activity was calculated by measuring the rate of increase in the absorption value.

## Results

### Structural and Phylogenetic Analysis of the *SlGATA17* Gene

We successfully cloned the full-length sequence of the CDS of *SlGATA17*, which was 100% consistent with the gene sequence in the Solanaceae Genomics Network. The gene was composed of 1,084 bases and encoded 327 amino acids. Phylogenetic relationships were analyzed for all the GATA family genes in tomatoes, and the GATA family genes of tomatoes were divided into four groups. *SlGATA17* belonged to the first group (Supplementary Figure S1).

### Expression Patterns of the *SlGATA17* Gene Under Different Exogenous Hormones

**FIGURE 1:**
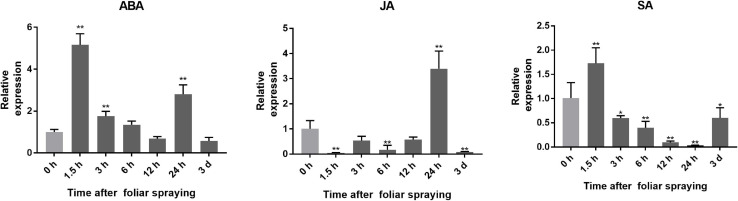
Expression of *SlGATA17* induced by exogenous hormones. Error bars represent the SD of triplicate experiments. Vertical bars with * and ** represent significant differences compared with 0 h, analyzed by one-way ANOVA (*P* ≤ 0.01**/0.05*).

The expression of *SlGATA17* at 1.5, 3, 6, 12, and 24 h and 3 days after ABA, JA, and SA foliar spraying is shown in Figure 1. *SlGATA17* was responsive to all three exogenous hormones, but there were differences in its response patterns to different hormones. After ABA and SA treatment, the expression of *SlGATA17* was upregulated rapidly in the short term. Then, the expression of *SlGATA17* began to decline over time, but the expression of *SlGATA17* did not drop below the initial level until 12 h after ABA treatment. However, the expression of *SlGATA17* dropped below the initial level at 3 h after SA treatment. After JA treatment, the expression of *SlGATA17* was downregulated and was below the initial level, except at 24 h. These findings indicated that the expression of *SlGATA17* had a strong response to ABA and SA in the early stage but not to JA.

### Analysis of TF Characteristics

**FIGURE 2:**
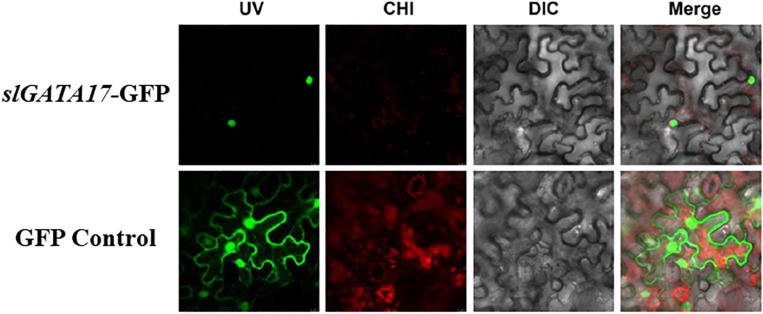
Subcellular localization of *SlGATA17* in tobacco leaf epidermal cells. The four images in each row from left to right are the green fluorescence signal, red fluorescence signal, light-field images, and merged images of the first three.

**FIGURE 3:**
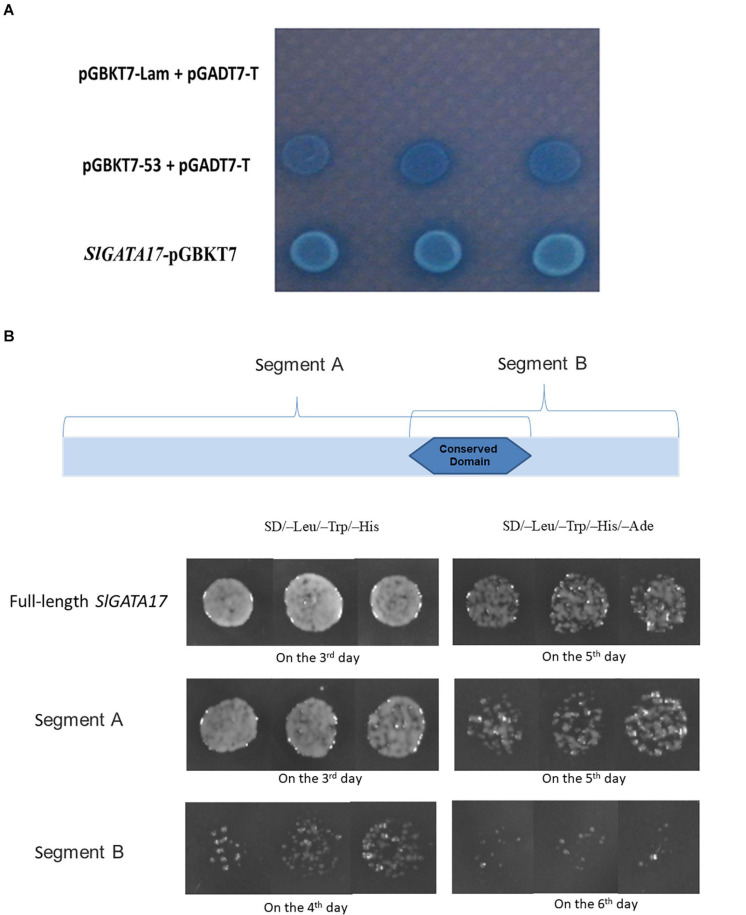
**(A)** Results of transformant growth on SD/–Leu/–Trp/–His with X-α-Gal medium. *SlGATA17* showed transcriptional activation activity. **(B)** Full length, segment A and segment B of *SlGATA17* transformants grown on SD/–Leu/–Trp/–His and SD/–Leu/–Trp/–His–Ade media. The picture shows that the transcriptional activation activity of segment A was stronger than that of segment B.

TFs are generally expressed in the nucleus and have transcriptional activation activity. To ascertain the subcellular localization of *SlGATA17*, we transformed the *SlGATA17*-GFP fusion protein and GFP protein into tobacco leaves. As shown in Figure 2, *SlGATA17*-GFP displayed strong fluorescent signals in the nucleus, whereas the control GFP signal was distributed throughout the cell. We cloned the full-length *SlGATA17*, the N-terminal segment of *SlGATA17* including the conserved domain (segment A), and the C-terminal segment of *SlGATA17* including the conserved domain (segment B). The activation activity of full *SlGATA17*, segment A, and segment B was evaluated using a GAL4 activation system. *SlGATA17*, segment A, and segment B fully activated the reporter genes in yeast (Figure 3). Same concentration and volume of transformants containing full length, segment A, and segment B of *SlGATA17* were spotted on SD/–Leu/–Trp/–His and SD/–Leu/–Trp/–His–Ade medium. The yeast clones containing segment A grew out earlier and more than those containing segment B (Figure 3B). These results suggested that the transcriptional activation activity of segment A is stronger than that of segment B.

### Generation of Transgenic Tomatoes

A total of eight tomato transgenic lines with kanamycin resistance were generated from tissue culture and detected by qPCR. Two independent transgenic lines (OE1 and OE5) were confirmed by qRT-PCR. The relative expression levels of *SlGATA17* were substantially higher in the OE1 and OE5 transgenic lines than in WT plants (Supplementary Figure S2). The two lines at T2 and subsequent generations were used for further analysis.

### Phenotypic Identification Results of Transgenic Plants Under Drought Treatment

**FIGURE 4:**
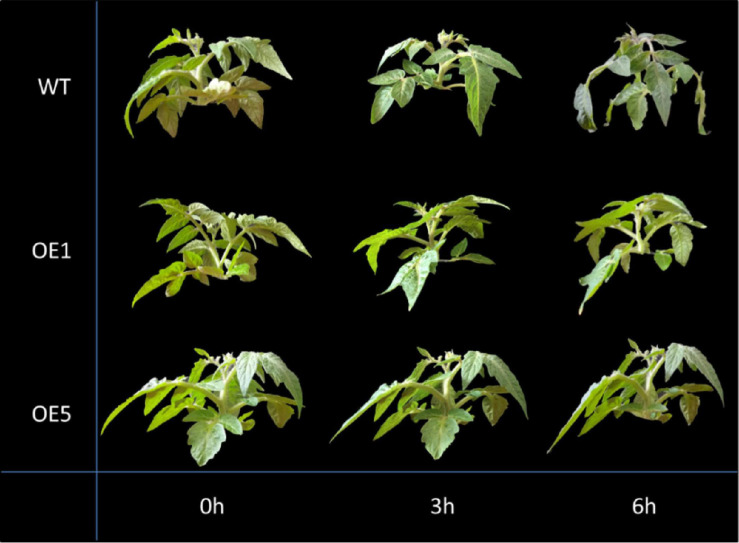
Phenotype comparison of representative *SlGATA17* transgenic tomato plants and WT plants under drought stress.

After 3 h of drought treatment, the leaf margins of WT plants were slightly curled, but OE1 and OE5 plants did not exhibit any change. After 6 h under drought treatment, WT plants showed the following changes: significant wilting, shriveled leaves, and drooping petioles. However, the leaves of the OE1 and OE5 plants only drooped slightly (Figure 4). These findings indicated that phenotypically, OE plants are more drought-tolerant than WT plants.

### Results of Physiological Index Measurements

**FIGURE 5:**
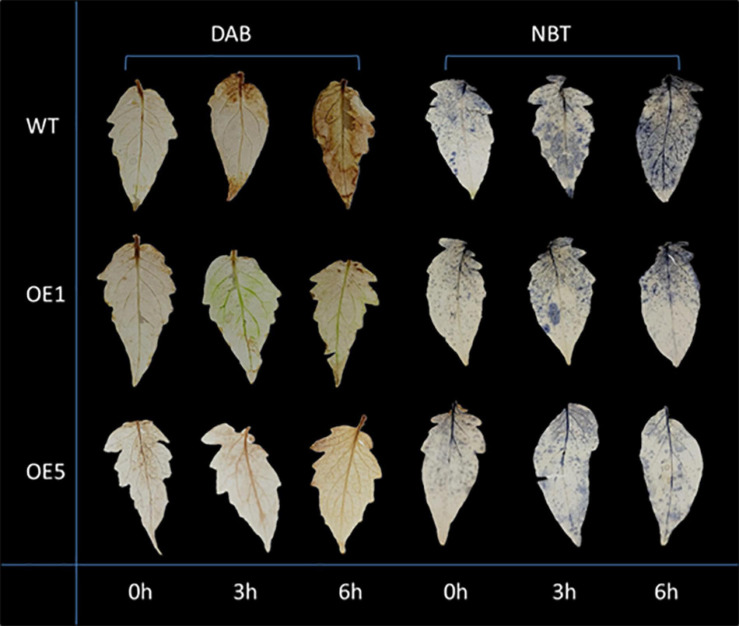
DAB and NBT staining of tomato leaves. The yellow and blue colors both represent superoxide accumulation.

**FIGURE 6:**
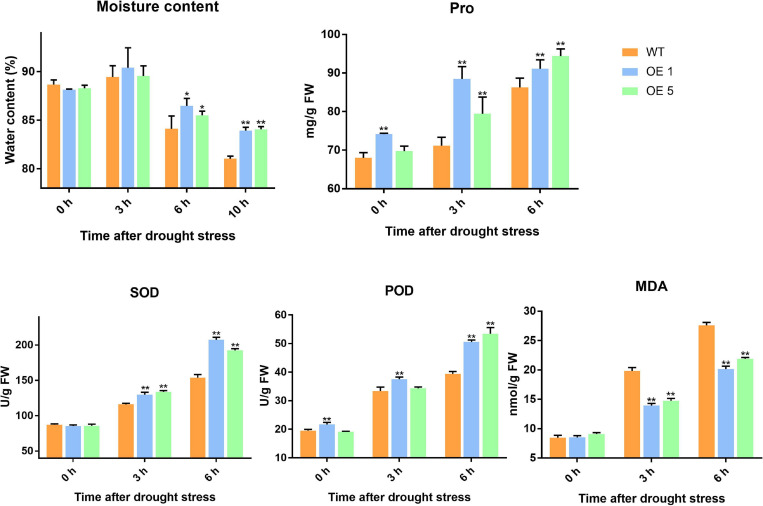
Water content, Pro content, SOD activity, POD activity, and MDA of WT and OE plants. Error bars represent the SD of triplicate experiments. Vertical bars with * and ** represent significant differences compared with WT at each time point, analyzed by one-way ANOVA (*P* ≤ 0.01**/0.05*).

Drought stress generally leads to ROS generation. Thus, DAB and NBT staining was used to detect the accumulation of H_2_O_2_ and O_2_^–^ (superoxide) radicals in leaves under normal and drought stress conditions. Under natural conditions, the difference in the activities of SOD between WT and transgenic plants was insignificant. After 3 and 6 h of drought stress, the amounts of brown precipitate (DAB staining) and blue color products (NBT staining) were greater in WT than in transgenic lines (Figure 5). The water content in the leaves of WT and OE plants increased slightly at 3 h of drought treatment, and there was no significant difference in water content at this time between WT and OE lines. At 6 h, however, the water content of WT and OE plants decreased significantly, and the decrease in water content in OE plants was greater. At 10 h after drought treatment, the difference in moisture content between the WT and OE lines was even more pronounced (Figure 6). As shown in Figure 6, the SOD activity, POD activity, and Pro content increased in both plants after drought stress, but they remained higher in OE plants than in WT plants. The MDA content increased in both WT and OE plants after drought stress but remained lower in OE plants than in WT plants. These results indicated that WT plants are more damaged by drought stress and that OE plants have a stronger ability to resist drought stress.

### Statistical Analysis of Transcriptome Sequencing Data

On average, 6.4 G of data was generated per sample. The details of raw reads and clean reads of each sample are shown in Supplementary Table S3. More than 96% of the clean reads had quality at the Q20 level, and more than 87% of the clean reads had quality at the Q30 level. In addition, 92% of the clean reads were mapped to the tomato reference genome.

### GO and KEGG Enrichment Analyses of the DEGs

**FIGURE 7:**
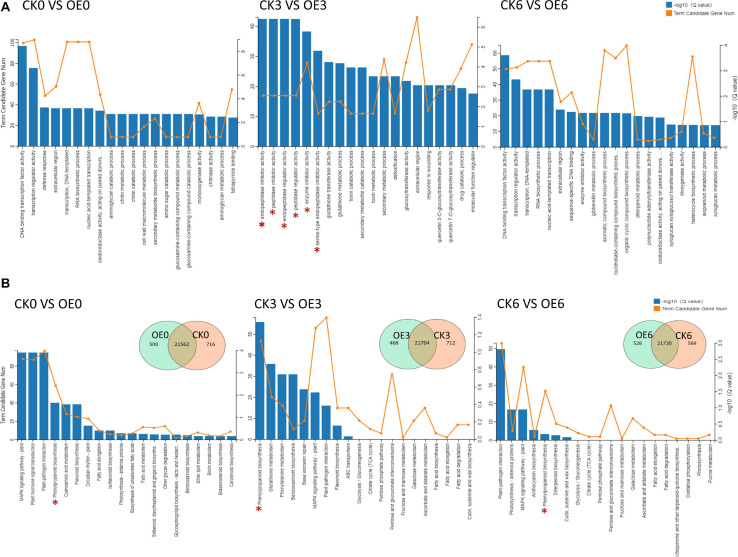
**(A)** GO annotation of DEGs at three time points. The asterisks represent the items of inhibiting or regulating protein degradation. **(B)** KEGG analysis and comparison of DEGs between WT and OE lines at three time points under drought stress. WT represents WT plants, and OE represents overexpression lines. The pentacle represents the phenylpropanoid biosynthesis pathway.

We conducted principal component analysis on six groups of triplicate RNA-seq data, and the results showed that there was a large difference between the OE plants and WT plants at 3 h after drought treatment (Supplementary Figure S3). DEGs were classified as those genes with an absolute log_2_ value greater than 1 based on RNA-seq results, and there were a large number of DEGs at all three time points. To investigate the biological significance of the genes regulated by drought stress in tomato, the DEGs were annotated by GO. DEGs were enriched in multiple GO terms at the three time points. At 3 h under drought treatment, many DEGs were enriched in endopeptidase inhibitor activity, peptidase inhibitor activity, endopeptidase regulator activity, peptidase regulator activity, and enzyme inhibitor activity items (Figure 7A). In the comparison between the CK3 and OE3 groups, many DEGs were enriched in the phenylpropanoid biosynthesis pathway and pathways related to the phenylpropanoid biosynthesis pathway (phenylalanine metabolism pathway and flavonoid biosynthesis pathway). In addition, DEGs at all three time points were enriched in phenylpropanoid biosynthesis pathways. Two common pathways, the MAPK signaling pathway and plant–pathogen interaction, were enriched by DEGs at all three time points (Figure 7B). The pathways with *Q* < 0.05 were further screened out in the comparison of the three time points. A schematic diagram of the differential regulation process at different stages is shown in Supplementary Figure S4. The overexpression of *SlGATA17* directly led to changes in the expression of related genes in the MAPK signaling pathway, plant hormone signal transduction, and phenylpropanoid biosynthesis. The differential expression of the phenylpropanoid biosynthesis pathway was further strengthened after drought stress. Finally, after 6 h under drought stress, some genes that respond to biotic stresses were enriched.

### Phenylpropanoid Biosynthesis Pathway Analysis

**FIGURE 8:**
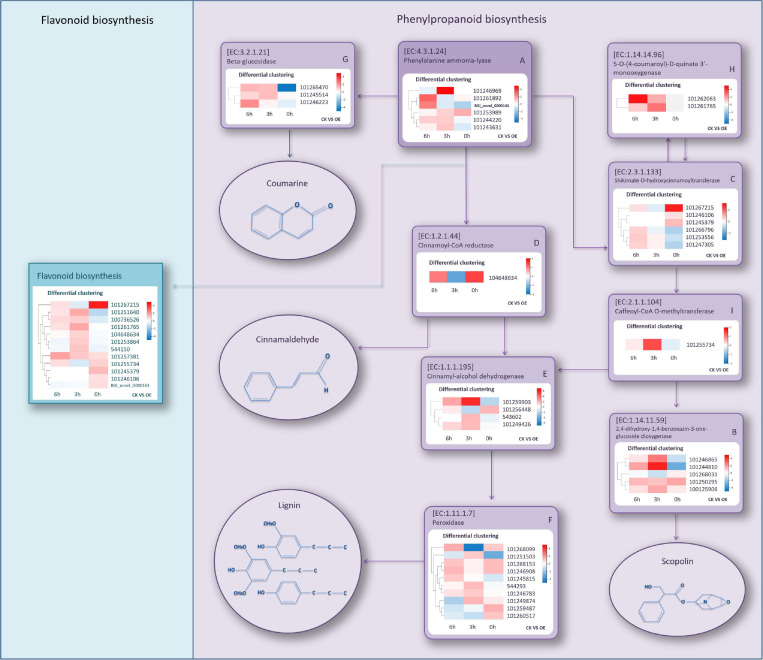
Simplified schematic diagram of regulatory points with upregulated genes encoding various enzymes and heatmaps of differential expression of these genes. The compounds in the circles are the metabolites catalyzed by these enzymes.

As the most obvious DEG-enriched pathway, we analyzed the phenylpropanoid biosynthesis pathway in depth. Gene expression of WT and OE lines in this pathway at three different time points was compared, and we selected all the gene regulatory points containing upregulated expression in the three groups. At 0 h, a total of six (A–F) regulatory points contained upregulated genes encoding various enzymes in the OE lines (Supplementary Figure S5). At 3 h, upregulated genes appeared at three new regulatory points, namely, G, H, and I (Supplementary Figure S6). However, at 6 h, there were no additional new regulatory points containing upregulated genes. Therefore, a total of nine regulatory points were screened out, including the key rate-limiting enzyme, PAL, in this pathway (EC: 4.3.1.24) and eight other regulatory points related to the synthesis of coumarin, lignin, cinnamaldehyde, and scopolin. These regulatory points were positively regulated by the *SlGATA17* gene. A simplified regulatory relationship schematic diagram of these regulatory points is shown in Figure 8. DEGs with higher expression levels in OE lines than in WT lines at any time point within the three were selected. The heatmap of differential expression is shown in Figure 8. Regulatory points A and F had the largest number of DEGs (10 and 6, respectively). There was only one gene at the D and I regulatory points. Most of the genes showed more than twofold change expression levels at two or three time points. After 3 h, the number of genes that had higher expression levels in the OE plants than in the WT plants increased, indicating that most of the genes had significantly upregulated responses after drought stress. The flavonoid biosynthesis pathway, as a phenylpropanoid biosynthesis downstream metabolic pathway, was relatively active in the OE lines and was also the significantly DEG enriched pathway at 0 h. Among all 12 DEGs, there were six DEGs with higher expression levels in OE lines than in WT lines at 0 h, and the other six DEGs had higher expression levels in OE lines than in WT lines at 3 h. These results indicated that genes in this pathway are regulated by *SlGATA17* in different ways.

### Analysis Results of PAL Activity

**FIGURE 9:**
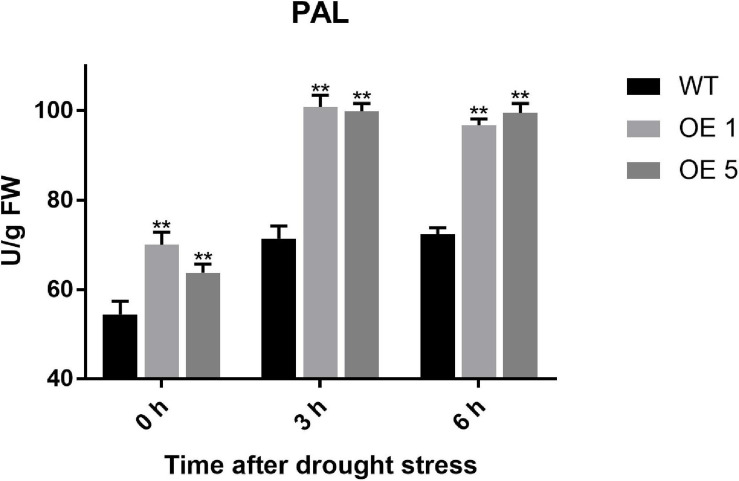
PAL activity of WT and OE plants. The PAL activity of OE lines was significantly higher than that of WT plants after drought treatment. Vertical bars with * and ** represent significant differences compared with WT at each time point, analyzed by one-way ANOVA (*P* ≤ 0.01**/0.05*).

To further verify the initial activity of the phenylpropanoid biosynthesis pathway, PAL activity, the first and most important catalytic enzyme in the phenylpropanoid biosynthesis metabolic pathway, was detected. At 0 h, the PAL activity of OE plants was significantly higher than that of WT plants. At 3 h under drought stress, the PAL activity of OE1, OE5, and WT plants all increased and maintained nearly the same level until 6 h after drought stress. However, the PAL activity of WT plants was considerably less active than that of OE1 and OE5 plants (Figure 9). This suggested that overexpression of *SlGATA17* activates the enzyme activity that initiates this pathway.

### Validation of RNA-Seq Data via qRT-PCR

To validate the accuracy of the data obtained from RNA-seq, 15 DEGs were chosen randomly for confirmation by qRT-PCR at three different time points. The 15 pairs of primers used for validation of RNA-seq are shown in Supplementary Table S2. Pearson correlation coefficients between the RNA-seq and qRT-PCR results of each gene at three time points in WT and OE plants are shown in Supplementary Figure S7. These results were largely consistent with the RNA-seq results, thereby validating the RNA-seq results.

## Discussion

GATA TFs are evolutionarily conserved transcription regulators that recognize promoter elements with an (A/T)G-A-T-A (A/G) amino acid sequence (Behringer and Schwechheimer, 2015). Although the roles of GATA proteins in photosynthesis (Chiang et al., 2012; An et al., 2020) and nitrogen metabolism (Berger et al., 2006) have been determined, their functions and the molecular mechanism of tolerance to abiotic stress have not been well studied to date. Furthermore, GATA proteins in tomato have rarely been studied for their biological functions. In this study, we functionally identified a tomato GATA gene family member, the *SlGATA17* TF, under drought stress.

GATA factors generally contain two zinc finger domains in animals (Katsumura et al., 2017). However, according to previous studies on plants, most GATA factors in plants have a single zinc finger domain (such as in *Arabidopsis*, rice, soybean, and grape) (Reyes et al., 2004; Zhang et al., 2015, 2018), which is consistent with the results of our *SlGATA17* protein sequence analysis. The transcriptional activation test indicated that the complete sequence of *SlGATA17* has self-activation ability. Further analysis of segment A of *SlGATA17* with an N-terminus containing a conserved domain and segment B of *SlGATA17* with a C-terminus containing conserved domain sequences showed that both ends have self-activating activity, but the self-activation ability of the N-terminus is stronger. We speculate that this is because the conserved domain common to segments A and B contains the transcriptional activation domain. This may also be due to the presence of other transcriptional activation domains in segment A or that there is less influence on the structure of the transcriptional activation domain in the non-conservative domain of segment A compared with the non-conservative domain in segment B.

Drought signal transduction is controlled by both ABA-dependent and ABA-independent pathways (Shinozaki and Yamaguchi-Shinozaki, 2000). To preliminarily determine whether the regulation of drought resistance by *SlGATA17* is related to ABA signaling, we analyzed the expression pattern of *SlGATA17* after endogenous ABA application and found that ABA significantly upregulated the expression of *SlGATA17*, indicating that the expression of *SlGATA17* is sensitive to ABA, which is likely to belong to the ABA-dependent regulation mode. In addition to ABA, SA and JA play an important role in the regulation of plant stress resistance and disease resistance. Methyl jasmonate has been found to improve resistance against abiotic stresses in many plants (Lee et al., 1996; Wang, 1999). SA induces several genes responsible for heat shock proteins, antioxidants, and secondary metabolites, thereby improving abiotic stress tolerance (Jumali et al., 2011). Therefore, we also analyzed the expression pattern of *SlGATA17* after induction of these two hormones and found that *SlGATA17* had a significant response to SA. The expression of this gene was significantly upregulated after induction of SA, indicating that the regulation of drought resistance by *SlGATA17* may also intersect with SA signals or be coregulated by ABA and SA.

To confirm the function of *SlGATA17* in the drought response of tomato plants, we successfully constructed overexpression lines of this gene. After simulating drought stress on the overexpression lines, we found that both phenotypic changes and changes in physiological indicators (moisture loss rate, ROS, MDA, SOD, POD, and Pro) showed that *SlGATA17* overexpression increased the drought resistance of tomato plants compared with the control plants, which confirmed that this gene is a positive regulatory gene for plants to respond to drought stress. The results of previous experiments have also shown that GATA TFs are related to enhancing plant tolerance to abiotic stress. Studies on the paralogous and functionally redundant GATA TFs in *Arabidopsis*, *GNC*, and *GNL/CGA1* have revealed that these two GATA TFs are involved in the process of improving cold tolerance (Richter et al., 2013). Overexpression of *OsGATA8* in rice improves tolerance to salinity and drought compared to WT plants (Nutan et al., 2020).

To further explore the ways in which the *SlGATA17* gene can improve the drought resistance of tomato plants, we performed transcriptome sequencing on the leaf samples of OE lines and WT plants before and after drought stress. KEGG analysis found that *SlGATA17* overexpression in the early stage under drought stress caused the phenylpropanoid synthesis pathway to be significantly enriched. The differential expression of genes at several downstream regulatory points of this pathway indicated that the *SlGATA17* gene was involved in the upstream regulation of the phenylpropanoid synthesis pathway.

KEGG analysis showed that phenylpropanoid synthesis was the most important differential metabolic pathway in drought resistance in this study. Phenylpropanoids are a large class of plant secondary metabolites. Phenylalanine is an aromatic amino acid, and phenylpropanoids are derived from phenylalanine in most plants. Phenylalanine can be deaminated by PAL to become cinnamic acid, which is further transformed to *p*-coumaric acid. Finally, *p*-coumaric acid is converted into *p*-coumaroyl-CoA under the catalysis of 4-coumaroyl CoA ligase (4CL), which is an important point in the pathway to generate various phenylpropanoid compounds (Vogt, 2010). *p*-Coumaric and *p*-coumaroyl-CoA eventually form lignin through multiple metabolic pathways (Supplementary Figure S5). Lignification in plants has been shown to increase drought tolerance in some studies, although the associated regulatory networks have not been well elucidated (Hu et al., 2009; Pereira et al., 2018). *p*-Coumaroyl-CoA is not only metabolized into lignin but also catalyzed by chalcone synthase to form chalcone and then further to form flavonoids after several steps (Supplementary Figure S5). Flavonoids, phenolic acids, and monolignols are the most common phenylpropanoids and are found in almost all terrestrial plants (Deng and Lu, 2017). Flavonoids and phenolic compounds in various plants are well-known potent antioxidants (Niggeweg et al., 2004; Deng and Lu, 2017). The flavonoid biosynthesis pathway, a phenylpropanoid biosynthesis downstream metabolic pathway, was also enriched at 0 and 3 h under drought stress, demonstrating that flavonoids are critically important metabolites for OE lines in response to stress resistance. Several studies have shown that flavonoids protect plants against a variety of biotic and abiotic stresses (Li et al., 1993; Simmonds, 2003; Rowan et al., 2009; Misra et al., 2010). Nakabayashi et al. (2014) confirmed that overaccumulation of flavonoids is key to enhancing drought tolerance in *Arabidopsis*. Ma et al. (2014) found that drought tolerance in wheat is related to the expression of flavonoid pathway genes and flavonoid compound accumulation. Many genes that encode metabolism-related enzymes at regulatory points are upregulated after drought stress in phenylpropanoid synthesis. The upregulation of these genes may increase the amount of the corresponding enzymes in the OE plants such that the corresponding metabolites increased. The increase in these substrates further activates the activity of the downstream flavonoid biosynthesis pathway. Therefore, we speculate that the *SlGATA17* gene enhances the drought tolerance of tomato plants by activating the expression of multiple genes involved in the synthesis of phenylpropanoids, especially flavonoids or lignin, thereby improving the antioxidant capacity through the accumulation of phenylpropanoids. PAL is a key enzyme in phenylalanine metabolism in plants. The increase in PAL activity under drought stress further corroborated the possibility of phenylpropanoid or lignin accumulation in *SlGATA17*-overexpressing plants.

At 3 and 6 h under drought stress, the expression levels of most genes related to PAL synthesis in OE plants were higher than those in WT plants. The PAL activity of OE plants was also significantly higher than that of WT plants at the two time points. However, there were only slight differences between the phenotype of OE plants and WT plants at 3 h. And there was no significant difference in water loss at 3 h. The phenotypic difference was very obvious at 6 h under drought stress, and the water loss was also significantly different at the same time. This suggests that phenotypic differences are later than changes in gene expression and enzyme activity.

The distribution of differential gene expression patterns at different regulatory points varied. For example, the upregulated expression of genes at the G and H points in OE lines occurred after 3 h relative to WT lines (Supplementary Figure S6). The upregulated expression of most genes at the F point appeared at 0 h, indicating that different regulatory points are regulated by the *SlGATA17* gene in different ways. Among the 12 DEGs in flavonoid biosynthesis, six DEGs showed upregulated expression at 0 h in OE lines relative to WT lines, and the other six DEGs showed upregulated expression at 3 h, indicating that the genes in flavonoid biosynthesis pathway are also regulated in different ways by *SlGATA17*.

The top five functional annotations of DEGs were all related to inhibiting or regulating protein degradation. This finding indicated that overexpression of the *SlGATA17* gene may prevent the degradation of some important functional proteins under drought stress or that the drought resistance mechanism is realized by improving posttranslational modification activities. These related proteins may play a direct role in resisting external stress or play a direct role as enzymes in regulating downstream metabolism, which are most likely related to the phenylpropanoid synthesis process. These possibilities warrant further research.

## Data Availability Statement

The original contributions presented in the study are publicly available. The data generated for this study can be found in the NCBI. The GEO accession number is GSE148530.

## Author Contributions

XX and TZ designed the study, supervised the project, and revised and drafted the manuscript. TW, TP, and ZW implemented the study and acquired the data. HY, JL, HZ, JJ, and XC helped draft the manuscript. All authors contributed to the article and approved the submitted version.

## Conflict of Interest

The authors declare that the research was conducted in the absence of any commercial or financial relationships that could be construed as a potential conflict of interest.

## Footnotes

1 http://smart.embl-heidelberg.de
