# Supplementary material for: Overexpression of SlGATA17 Promotes Drought Tolerance in Transgenic Tomato Plants by Enhancing Activation of the Phenylpropanoid Biosynthetic Pathway
Source: Front Plant Sci. 2021 Mar 16;12:634888. doi: 10.3389/fpls.2021.634888 (PMC8008128; doi:10.3389/fpls.2021.634888)
Supplement: Supplementary file 1 [file Data_Sheet_1.docx]

**Supplementary figures**


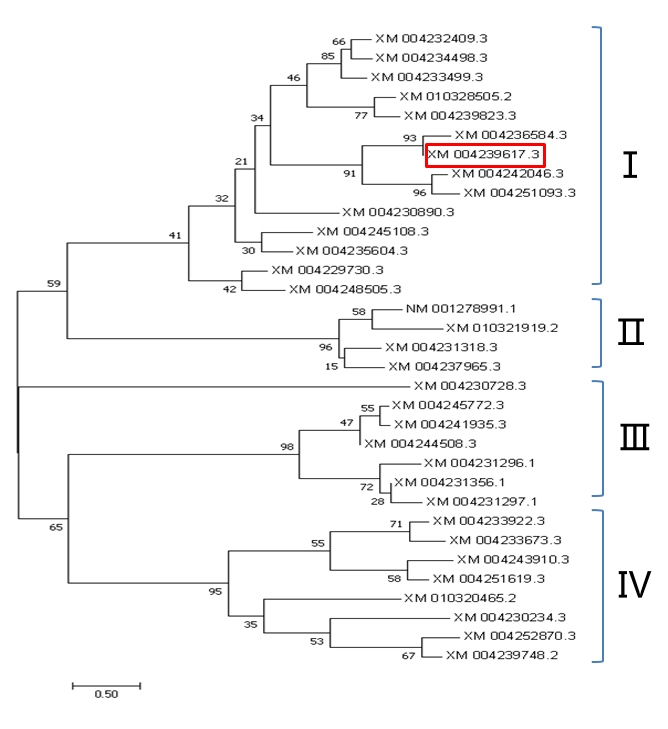


**Figure S1.** The phylogeny of GATA family genes in tomato. The gene in the red rectangle is *SlGATA17*.


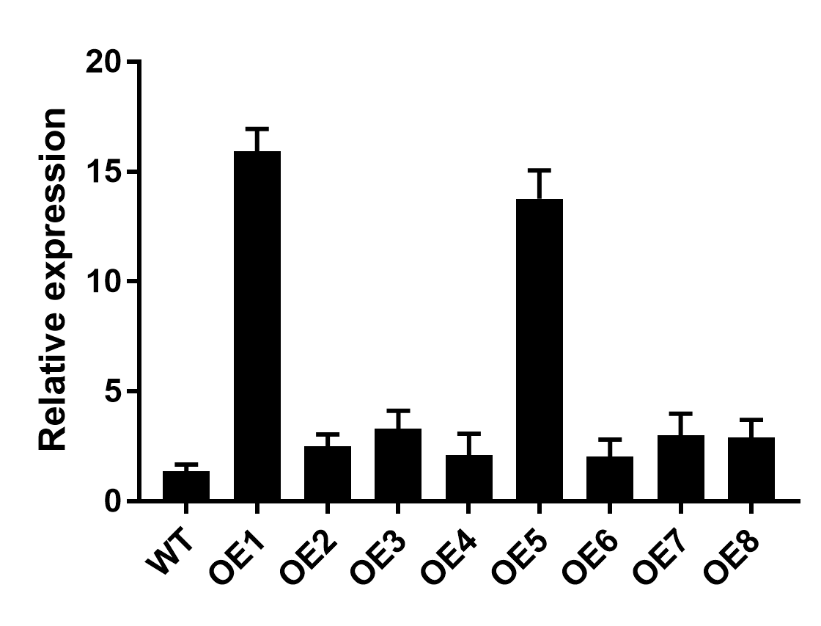


**Figure S2.** Identification of overexpression plants by qRT-PCR. Transcript levels of *slGATA17* in OE1 and OE5 transgenic lines were significantly higher than those in WT plants. Error bars represent the SD of triplicate experiments.


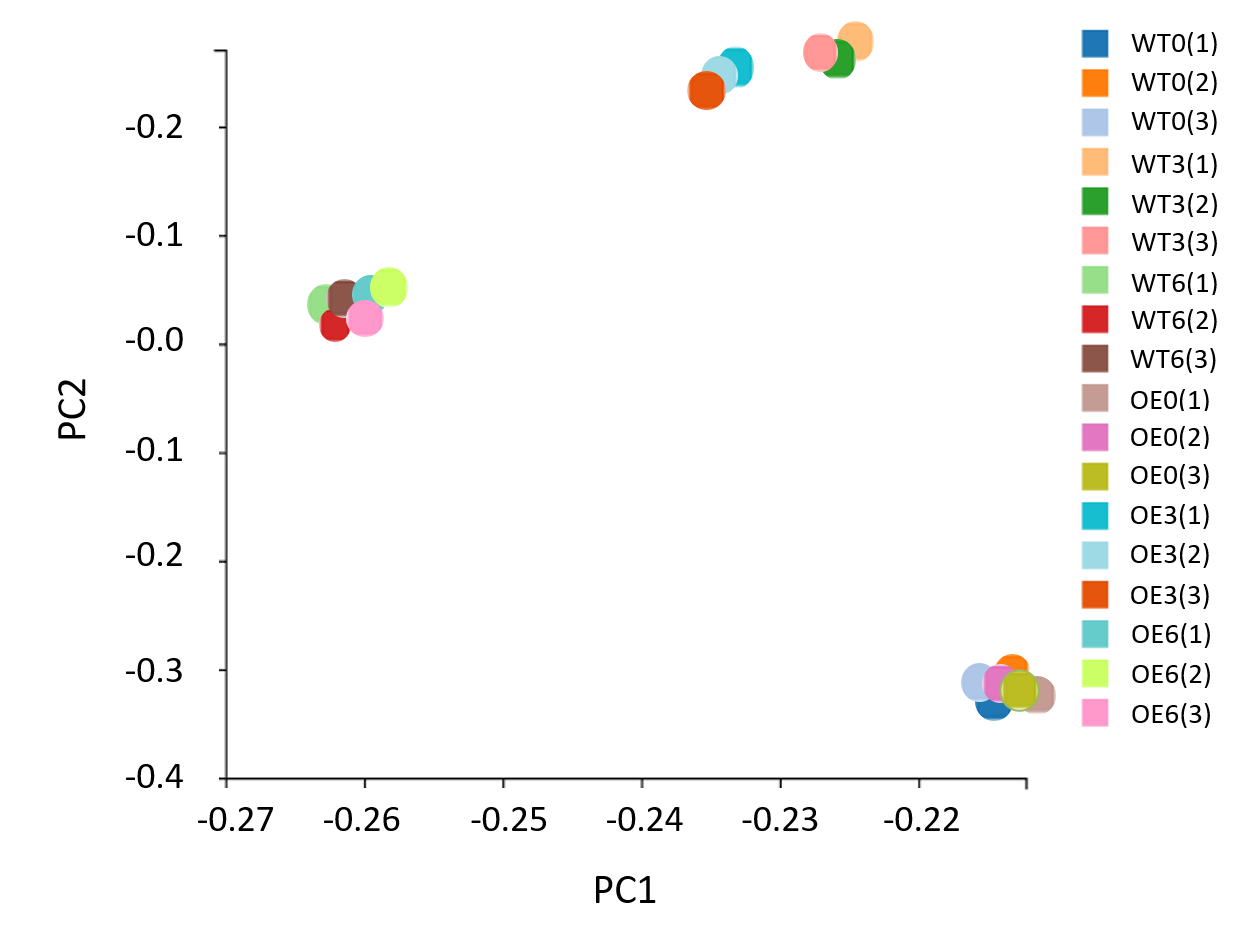


**Figure S3.** Principal component analysis of 6 groups of triplicate RNA-seq data. WT0, WT3 and WT6 represent WT plants under drought treatment at 0, 3 and 6 h, respectively. OE0, OE3 and OE6 represent OE plants under drought treatment at 0, 3 and 6 h, respectively.


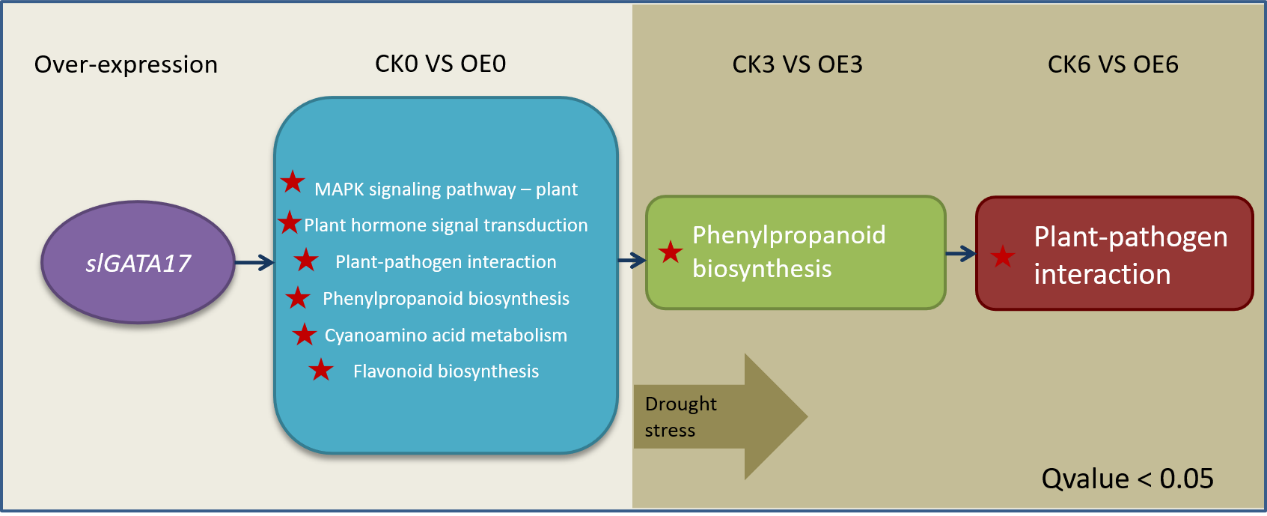


**Figure S4.** Changing process of pathway regulation from 0 h to 6 h under drought stress.


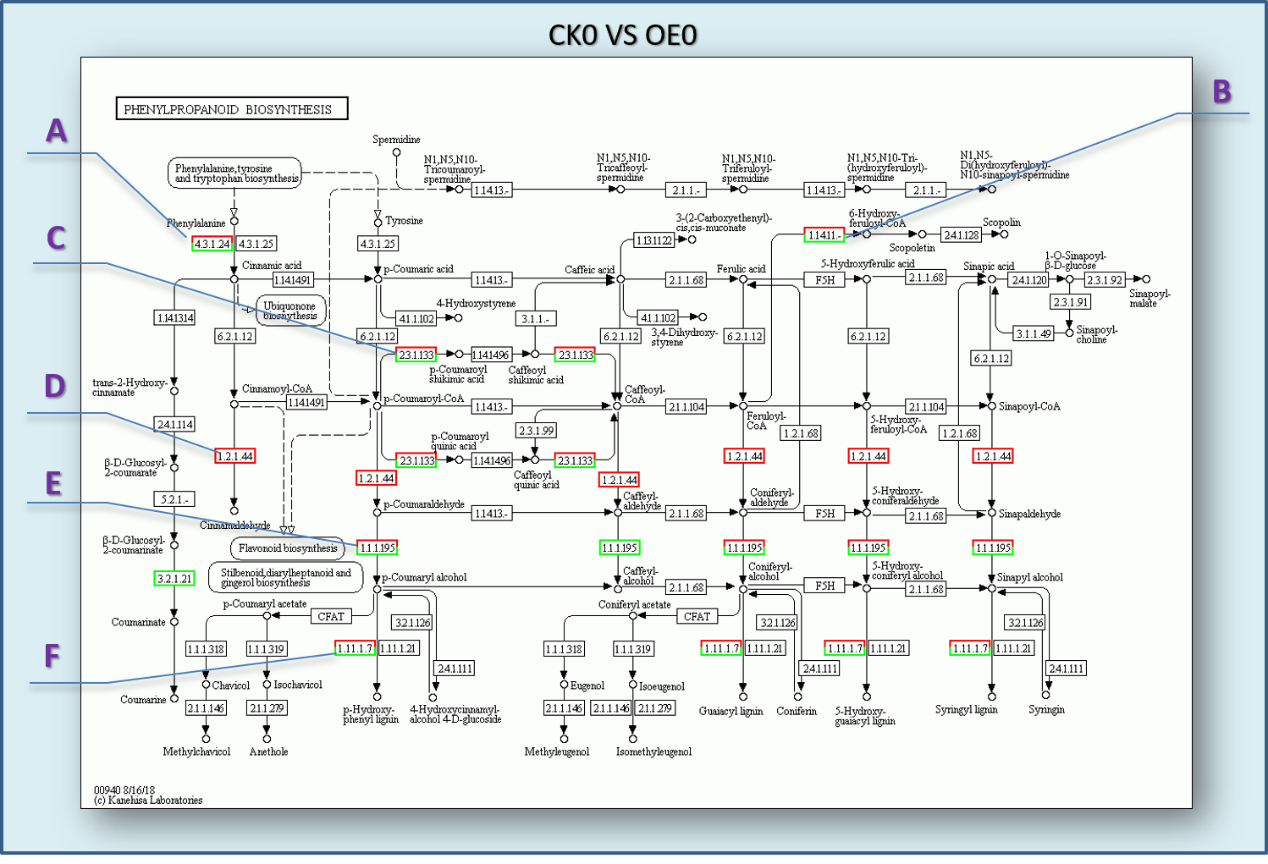


**Figure S5.** Phenylpropanoid biosynthesis metabolic pathway map at 0 h under drought stress. The red box represents upregulated genes of OE lines relative to WT lines at this regulatory point, and the green box represents downregulated genes at this regulatory point.


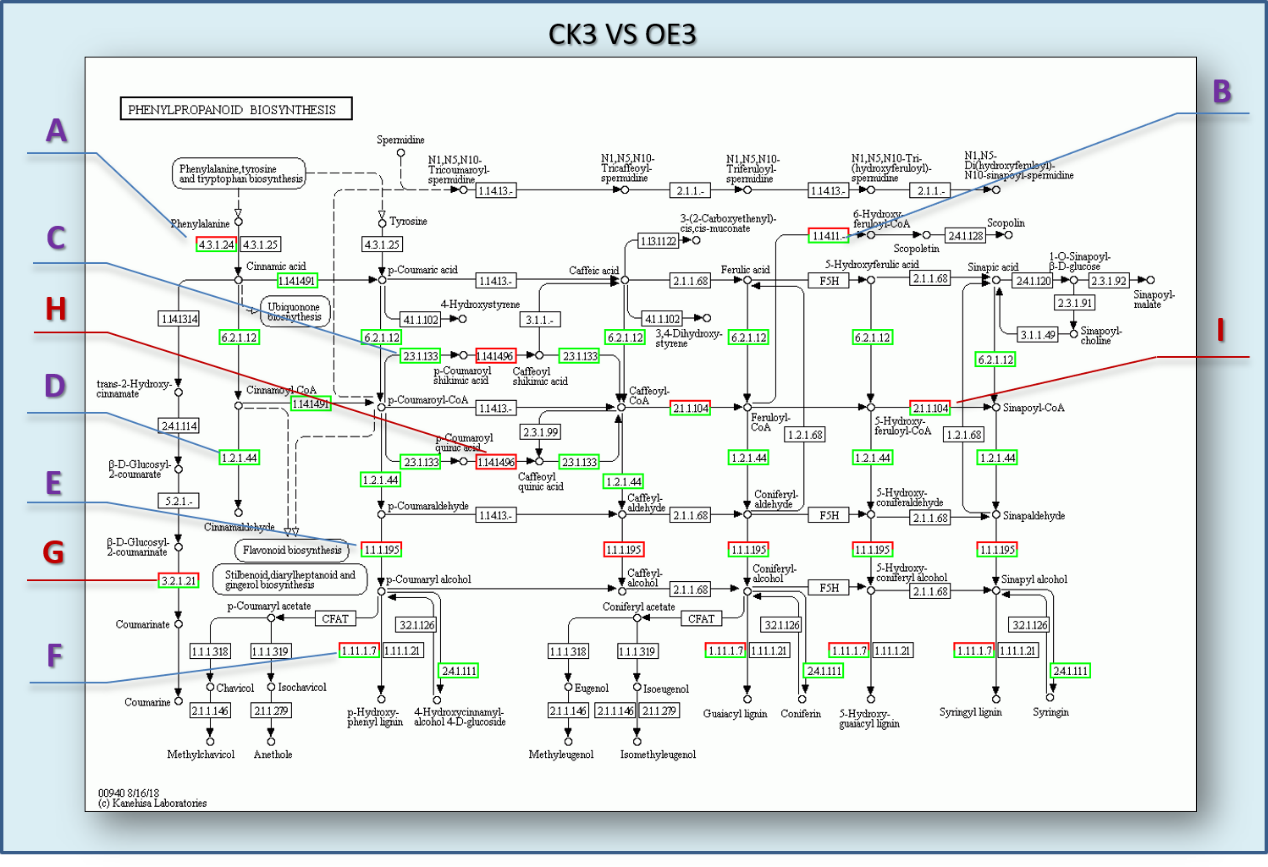


**Figure S6.** Phenylpropanoid biosynthesis metabolic pathway map at 3 h under drought stress. Compared with 0 h, there were three more regulatory points with upregulated expression genes.


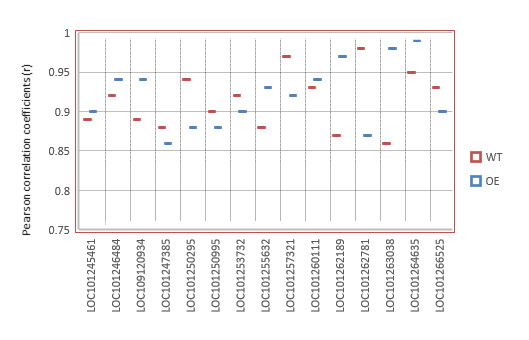


**Figure S7.** Pearson correlation coefficients between the RNA-seq and qRT-PCR results of each gene at three time points (0 h, 3 h and 6 h) in WT and OE plants.

**Supplementary tables**

**Table S1 Primers for gene cloning**

| *slGATA17*-GFP-F | GGGAAAATGGGGTCAAATGTGGTAGATG |
| --- | --- |
| *slGATA17*-GFP-R | CAGCTGCACAGCCAACTCCTCCTCGTGTTC |
| *slGATA17*-F | GGGAAAATGGGGTCAAATGTGGTAGATG |
| *slGATA17*-R | CAGCTGCACAGCCAACTCCTCCTCGTGTTCACT |
| A- *slGATA17*-F | GAATTCATGGGGTCAAATGTGGTAGAT |
| A- *slGATA17*-R | GGATCCTGGAACAAATGTAGGACTTGC |
| B- *slGATA17*-F | GAATTCGCAGTTCGGAAATGCCAGCA |
| B- *slGATA17*-R | GGATCCTCACTTGTGCTCCTCCTCAACC |

**Table S2 Primers for qRT-PCR**

| Gene | Primer-F | Primer-R | Product length |
| --- | --- | --- | --- |
| *slGATA17* | GAGTGAGTGGTGGTGACACTAA | GCTGCCAGAGAAAAGGGAGT | 412 |
| LOC101245461 | ATGTTGGTTGCTGAGAAAAATGAC | TTCCAAAAGTCGGAGTCAAGC | 189 |
| LOC101246484 | CAGCTCCCTCGCAGTTACAA | CGTGCATGCAAAATGCTTCC | 165 |
| LOC109120934 | AGCAGGCACCCATAAAGGAA | GGAACCTGCTTCACTGCCTA | 168 |
| LOC101247385 | TCAACACGTGACAGGCACAA | GCGTTGGACATTTTCCGGTG | 124 |
| LOC101250295 | AGGCTCGATCCGTCCATTTG | TTCCACGAGTCGAAAACCAA | 93 |
| LOC101250995 | AAGAGGGCTGTCCCTACCTC | AAGGCTTCGCTTTGGAGTCT | 166 |
| LOC101253732 | ACGTTGCAATGCCAACCATC | GGAGACGGCAACAAGGATGA | 255 |
| LOC101255632 | CCTCGGGCTTCTGTTTCAGT | GGGTCCTCAAACATCTGCAAG | 185 |
| LOC101257321 | ACCATCCTACCCACCCTTTT | ATTGCCTTAGCCTTCTTGGCT | 175 |
| LOC101260111 | TGAATAACATCAAGGAGGCGA | ACACGCGTTTCTCCCTCAAT | 367 |
| LOC101262189 | AAAACGAACCTCCCAAGGCA | CGCGTGAGCGATCTCTGTAT | 154 |
| LOC101262781 | CGGACGTATAAGCACCGTCA | CCTACCACTTGGAACCGACC | 171 |
| LOC101263038 | TCGGCAAAATCTCAGAAGAAGA | CACCAACACCACCGGTAGAA | 130 |
| LOC101264635 | CCCCGGAGGTGTTAGAATGG | TTTTGCGCGCCGGTATAATC | 103 |
| LOC101266525 | TGGAGTGCTGACTGTCACTG | CACTTGAATACAAAGACACAGCAAC | 166 |

**Table S2 Details of transcriptome sequencing of each sample**

| NO. | Sample | Total Raw Reads (M) | Total Clean Reads (M) | Total Clean Bases(Gb) | Clean Reads Q20(%) | Clean Reads Q30(%) | Clean Reads Ratio(%) |
| --- | --- | --- | --- | --- | --- | --- | --- |
| 1 | WT01 | 49.19 | 44.54 | 6.68 | 96.65 | 88.12 | 90.56 |
| 2 | WT02 | 47.43 | 43.23 | 6.48 | 96.67 | 88.17 | 91.15 |
| 3 | WT03 | 47.43 | 43.24 | 6.49 | 96.66 | 88.09 | 91.16 |
| 4 | WT31 | 47.43 | 43.57 | 6.54 | 96.53 | 87.75 | 91.86 |
| 5 | WT32 | 47.43 | 43.07 | 6.46 | 96.63 | 88.05 | 90.81 |
| 6 | WT33 | 47.43 | 43.71 | 6.56 | 96.7 | 88.19 | 92.16 |
| 7 | WT61 | 49.19 | 45.01 | 6.75 | 96.73 | 88.32 | 91.5 |
| 8 | WT62 | 47.43 | 43.64 | 6.55 | 96.73 | 88.33 | 92 |
| 9 | WT63 | 47.43 | 43.42 | 6.51 | 96.75 | 88.36 | 91.56 |
| 10 | OE01 | 47.43 | 43.12 | 6.47 | 96.73 | 88.34 | 90.91 |
| 1 | OE02 | 50.94 | 46.31 | 6.95 | 96.73 | 88.31 | 90.89 |
| 2 | OE03 | 47.43 | 43.15 | 6.47 | 96.63 | 88.07 | 90.98 |
| 3 | OE31 | 47.43 | 43.82 | 6.57 | 96.62 | 87.99 | 92.39 |
| 4 | OE32 | 43.8 | 40.74 | 6.11 | 96.75 | 88.36 | 93.03 |
| 5 | OE33 | 49.19 | 44.83 | 6.73 | 96.75 | 88.33 | 91.15 |
| 6 | OE61 | 49.19 | 44.9 | 6.73 | 96.69 | 88.17 | 91.28 |
| 7 | OE62 | 49.19 | 45.05 | 6.76 | 96.79 | 88.45 | 91.59 |
| 8 | OE63 | 47.43 | 43.6 | 6.54 | 96.48 | 87.6 | 91.92 |
